# Pharmaceutical Oral Formulation of Methionine as a Pediatric Treatment in Inherited Metabolic Disease

**DOI:** 10.3390/pharmaceutics15030957

**Published:** 2023-03-16

**Authors:** Benjamin Querin, Arnaud Schweitzer-Chaput, Salvatore Cisternino, Sylvain Auvity, Anne-Sophie Fauqueur, Abdel Negbane, Alice Hadchouel, Joël Schlatter, Camille Cotteret

**Affiliations:** 1 Service Pharmacie, Hôpital Universitaire Necker—Enfants Malades, Assistance Publique des Hôpitaux de Paris (AP-HP), 149 rue de Sèvres, F-75015 Paris, France; 2 Université Paris Cité, Inserm UMRS 1144, Faculté de Pharmacie, 4, Avenue de l’Observatoire, F-75006 Paris, France; 3 Service de Pneumologie Pédiatrique, Hôpital Universitaire Necker—Enfants Malades, Assistance Publique des Hôpitaux de Paris, AP-HP, 149 rue de Sèvres, F-75015 Paris, France; 4 Institut Necker Enfants Malades (INEM), INSERM U1151, Faculté de Médecine, Université Paris Cité, 156 rue de Vaugirard, F-75015 Paris, France; 5 Service Pharmacie, Hôpital Paul Doumer, Assistance Publique des Hôpitaux de Paris, AP-HP, 1 rue de l’hôpital, F-60140 Labruyère, France

**Keywords:** acceptability, methionine, nutrient, rare disease, stability indicating

## Abstract

L-Methionine (Met) is an essential alpha-amino acid playing a key role in several metabolic pathways. Rare inherited metabolic diseases such as mutations affecting the MARS1 gene encoding methionine tRNA synthetase (MetRS) can cause severe lung and liver disease before the age of two years. Oral Met therapy has been shown to restore MetRS activity and improve clinical health in children. As a sulfur-containing compound, Met has a strongly unpleasant odor and taste. The objective of this study was to develop an optimized pediatric pharmaceutical formulation of Met powder, to be reconstituted with water, to obtain a stable oral suspension. Organoleptic characteristics and physicochemical stability of the powdered Met formulation and suspension were evaluated at three storage temperatures. Met quantification was assessed by a stability-indicating chromatographic method as well as microbial stability. The use of a specific fruit flavor (e.g., strawberry) with sweeteners (e.g., sucralose) was considered acceptable. No drug loss, pH changes, microbiological growth, or visual changes were observed at 23 ± 2 °C and 4 ± 2 °C with the powder formulation for 92 days, and the reconstituted suspension for at least 45 days. The developed formulation facilitates the preparation, administration, the dose adjustment and palatability of Met treatment in children.

## 1. Introduction

L-methionine (Met) is a vital sulfur-containing α-amino acid playing a key role in mammalian metabolism such as methyl donor reactions, as well as diverse cellular functions such as mRNA translation and protein synthesis [1]. Although various biochemical pathways involve Met, it is not synthesized in the body and its supply from diets is critical. Met is the substrate of the S-adenosylmethionine (SAM) synthetase, an enzyme which combine both Met and ATP to form SAM, a major methyl donor [1,2]. After demethylation or hydrolysis, SAM is metabolized into homocysteine and possibly regenerated after homocysteine methylation by the Met synthase and the 5,10-methylenetetrahydrofolate reductase (MHTFR) [1,2]. Although Met-related biochemicals such as SAM have major cellular roles, Met is also a key cellular component in the mRNA translation. Indeed, to initiate translation, Met is directly attached to tRNA by the methionine tRNA synthetase (MetRS; EC 6.1.1.10), leading to the formation of methionyl-tRNA. MetRS is encoded by the *MARS1* gene. This methionyl-tRNA allows the initiation of translation, by appropriate positioning at the initiation AUG codon of the mRNA, a 40S ribosomal subunit containing the initiator methionyl-tRNA and a 60S ribosomal subunit binding to form the 80S complex to start initiation and the transition to elongation [3,4].

Diseases caused by variants of *MARS1* have been described such as the Charcot-Marie-Tooth disease type 2, a group of peripheral nervous hereditary disorders involving sensory and motor peripheral nerves dysfunctions [5]. Another inherited human disease affecting MetRS has been described, caused by an autosomal recessive inheritance. In particular, a cluster of patients from the island “La Réunion” was characterized by the double homozygous Ala393Thr/Ser576Leu mutation (#615486 in OMIM web database) on *MARS1* [6]. This specific mutation is located in the catalytic MetRS site, causing an impairment in enzymatic activity with a decrease of the catalytic enzyme activity (kcat) by 5- to 6-fold as compared to the wild-type protein [7]. This mutation led to a specific clinical phenotype named as the interstitial lung and liver disease (ILLD). It is characterized by systemic inflammation, a liver disease including elevated liver enzymes, steatosis, fibrosis and sometimes cirrhosis, and a specific type of pulmonary alveolar proteinosis (PAP). 

The particular type of PAP caused by mutations in the *MARS 1* gene has been described as being severe and usually manifesting in early childhood. It included dyspnea, cough, digital clubbing and severe respiratory distress often leading in death in childhood due to lung fibrosis, reaching 59% with half of them occurring before the age of two years [7]. Therapeutic management consisted mainly of poorly tolerated whole lung alveolar lavage and high-dose steroid therapy until, more recently, the benefits of Met therapy were noted [6]. Indeed, based on previous in vitro studies performed in yeast expressing or not expressing the mutated MARS1 gene, Met supplementation showed a restoration of the enzymatic MetRS activity, also confirmed in recent clinical studies performed in treated patients aged from 6- to 35-months old [6,7]. Daily oral Met supplementation was provided four times per day by oral or enteral administration of a pure Met powder diluted extemporaneously in water. Met dosing started at 80 mg/kg/day and progressively increased until reaching plasma concentrations between 45 and 500 µM at residual and peak states, respectively. This treatment resulted in significant improvements in clinical, biochemical, and imaging parameters [6].

To our knowledge, there is currently no validated pediatric pharmaceutical form of Met for the oral route. Given the young age of patients, we aimed to develop a pharmaceutical liquid form of Met. A liquid dosage form facilitates administration to young children and adjustment of the dose, and improves the acceptability of the treatment [8,9]. Being a sulfur amino acid, Met exhibits a strong unpleasant sulfur smell and taste, which can be attenuated with an appropriate pharmaceutical formulation such as the addition of flavors and sweeteners, and complexation strategies such as cyclodextrin, lipophilic vehicles, or microencapsulation [10,11,12,13,14]. In addition, given the tropical regions where most patients are located (e.g., the islands of Reunion, Comoros, and Madagascar), we sought to maximize stability and provide an easily reproducible formulation. We aim to develop a powder mixture to maximize stability and providing an oral suspension after reconstitution when needed.

## 2. Materials and Methods

### 2.1. Drugs and Chemicals

Pharmaceutical grade Met pure powder was obtained from Fagron (Thiais, France), sodium benzoate and xanthan gum pharmaceutical pure powders were obtained from Cooper (Melun, France). Pharmaceutical sodium carmellose, sodium citrate, and citric acid were supplied by Caelo (Hilden, Germany). Sucralose was provided by Inresa pharmaceutical (Bartenheim, France). Strawberry, orange, litchi, and candy floss flavors were purchased from Scrapcooking (Tours, France). Acetonitrile and orthophosphoric acid were of analytical grade, and purchased from VWR chemicals (Fontenay-sous-Bois, France). Distilled sterile water was purchased from Aguettant (Lyon, France).

### 2.2. Validation of Stability-Indicating Liquid Chromatography Met Assay

#### 2.2.1. Equipment and Analytical Conditions

Met assay was performed by a high-performance liquid chromatography (HPLC) system (Dionex, Ultimate 3000, Thermo Scientific, Villebon-sur-Yvette, France) with HPG-3200SD quaternary pump and WPS-3000TSL autosampler, coupled to a Dionex MWD-3000 diode array detector (DAD). HPLC system data acquisition (e.g., peak time, area under peak) was carried out using the Chromeleon^®^ software (v6.80 SP2, Thermo Scientific). A Polaris^®^ C18 column (250 × 4.6 mm; particle size, 5 µm; Agilent, Les Ulis, France) maintained at 30 °C using a column heater was used. The mobile phase consisted of acetonitrile and water (0.5/99.5 *v*/*v*) adjusted to pH 2.1 with orthophosporic acid. The column temperature, the mobile phase, the flow rate, the injection volume and the detection for detection and quantification were set at 30 ± 2 °C, 1.0 mL/min, 30 µL and 205 nm, respectively.

#### 2.2.2. Validation of the HPLC Assay Method

The method was validated according to the guidelines by assessing the linearity, accuracy, specificity, and precision [15]. Two stock solutions of Met were prepared daily over three days by diluting Met in distilled water to a final concentration of 1 mg/mL, to independently prepare five calibration standard (between 160 and 240 µg/mL) and three quality controls (170; 190; and 230 µg/mL, recorded in triplicate). Each day, the slope, intercept and correlation coefficient (r^2^) were calculated to study linearity. Accuracy was determined using quality controls, and expressed as the percentage of recovery determined by the following equation: 

$\frac{\mathrm{experimental}\;\mathrm{concentration}}{\begin{array}{c} \mathrm{Theoritical}\;\mathrm{concentration} \end{array}}\times 100$, with ± 5% as acceptance criteria. Matrix effect was assessed by comparing calibration curve of Met in water and in presence of all formula excipients.

Precision of the method was studied using quality controls recorded in triplicate on three independent days. The intra-days analysis was performed by calculating the relative standard deviation (RSD) of calculated compared to theoretical concentration on each quality control recorded in triplicate the same day (*n* = 3). The inter-days analysis was performed by calculating the RSD on each quality control recorded over three days (*n* = 9). Additionally, repeatability was estimated by recording 10 times a 200 µg/mL Met solution and calculating the RSD. 

The limit of detection (LOD) and limit of quantification (LOQ) for Met were evaluated based on response standard deviation and calibration curve slope based on the following equations: LOD = $3.3\times \frac{s\left(\mathrm{intercept}\right)}{s\left(\mathrm{slope}\right)}$; LOQ = $10\times \frac{s\left(\mathrm{intercept}\right)}{s\left(\mathrm{slope}\right)}$, where s(intercept) and s(slope) are the standard deviation of the y-intercept and slope of the calibration curve, respectively.

#### 2.2.3. Selectivity of the Met Analytical Method

The selectivity of the method was ensured so that no chromatographic peak of an excipient or degradation product coincided with that for Met. Chromatograms of Met in the presence of all formula excipients were visually inspected to detect changes in shape of the Met peak as well as by the analysis of the Met peak UV spectra recorded by DAD (200–300 nm).

Then, four stress conditions were applied on the Met formula described in Table 1: acidic (1 M HCl), alkaline (1 M NaOH) and oxidative (0.03% H_2_O_2_) stress conditions for 72 h (H72) at 70 °C, and photolytic stress for 32 h (H32) with samples taken and analyzed at various time points. To perform the forced degradation study, a stock solution of Met with excipients (see Table 1) was prepared (200 mg/mL of Met). This solution was then diluted in water to a final concentration of 400 µg/mL of Met, and finally diluted in equal parts with either aqueous 1 M HCl, 1 M NaOH or 0.03% H_2_O_2_, and ultrapure water. Photolytic stress studies were performed with a QSun-Xe-1 device (Q-Lab Corporation, Saarbrücken, Germany) equipped with a xenon lamp and a daylight-Q filter (Q-Lab), which simulates natural sunlight (wavelength from 300 to 800 nm) operating in window mode and complying with ICH Q1B recommendations. The light beam obtained simulate CIE Standard Illuminant D65 (Q-Lab), with irradiance set at a constant intensity constant (1.5 W/m^2^ 420 nm). The temperature was controlled and set at 23 ± 2 °C.

### 2.3. Composition of the Met Powder Formulation

A powder mixture ready to be reconstituted with water has been formulated to obtain a suspension of Met at 200 mg/mL suitable for an oral administration in children. It was composed of a mix of pharmaceutical grade Met, and selected pharmaceutical grade anhydrous excipients such as preservative (sodium benzoate), viscosifier and thickener (e.g., sodium carmellose, xanthan gum), pH buffer (sodium citrate and citric acid), sweetener (sucralose), and aroma (e.g., fruit flavor).

To make the taste and smell of the preparation more attractive, a fruit flavoring has been added in a selected amount: orange, cotton candy, lychee and strawberry. Five adults rated the Met formulas containing the selected fruit flavor on a subjective scale from 1 to 5, 5 being the best score for taste. Different amounts of sucralose (0.05–0.2% *m*/*v*) were tested by the same taste scoring methodology.

Visual inspection of Met suspensions containing various amounts of xanthan gum (0.3–0.5% *w*/*v*) was performed to assess particle distribution and agglomeration. The uniformity of Met content was determined using two methods. First, after 10 s of manual bottle shaking, samples were taken (in triplicate) at three sampling levels: at the bottom, middle, and top of the suspension. These samples (*n* = 3) were analyzed for Met content by HPLC assay. Secondly, suspension samples (1 mL; *n* =3) were withdrawn with an oral syringe (Nutrisafe^®^, Vygon, Ecouen, France) after manual bottle shaking for 10 s, and at different times after water reconstitution of the bulk powder: 1 h, 5 h, and 3 days. All samples were analyzed for Met content using the HPLC assay.

Viscosity of the Met suspension was evaluated at 25 °C using a calibrated viscosity cup (Zahn, diameter 5.3 mm, VWR, Rosny, France) according to the manufacturer’s instructions. Briefly, the cup is immersed in the suspension at 25 °C until it is filled, and the time needed to leave the surface of the liquid until the first break point in flow is monitored. The kinetic viscosity is then calculated and transformed by mass volume correction to obtain absolute viscosity.

The final optimized composition of the Met formulation was detailed in Table 1 and was used in all other Met stability studies.

To prepare one container at 200 mg/mL Met powder oral formulation, ingredients are exactly weighed as described in Table 1. Powders were mixed and transferred into a 200 mL volume transparent type 3 glass bottle obtained from SGD Pharma (Sucy en Brie, France). For reconstitution, 100 mL of water is added in two equal parts (e.g., two times 50 mL) and shaken vigorously for 1 min after the first half addition of water. At the end, the mixture is shaken vigorously for one min.

### 2.4. Physicochemical Stability Experiments

Met stability studies were performed with the Met formula shown in Table 1 stored at 4 ± 2 °C (17 ± 3% relative humidity), 23 ± 2 °C (55 ± 5% relative humidity), and 40 ± 2 °C (10 ± 1% relative humidity). At day 0, three independent preparations were made and stored in each storage conditions. The Met concentration was calculated every day using a calibration curve made on the same day and validated by three quality controls prepared independently. The remaining Met concentration was calculated by the ratio of the concentration on the tested day to the concentration at day 0 (D0). Preparations were considered stable if organoleptic aspects were unchanged and Met concentration remained above 90% of the initial concentration.

#### 2.4.1. Unreconstituted Met Formulation: Stability Experiments over Time

Powder samples of each preparation were analyzed on days 0, 3, 7, 14, 21, 30, 45, 60, 75 and 90. After shaking, 200 mg of each sample were diluted into a 1000 mL volumetric flask with distilled water and mixed for 10 min before being analyzed by HPLC.

#### 2.4.2. Water Reconstituted Met Formulation: Stability Experiments over Time

Following water addition and shaking (D0; day 0), aliquots of suspension from each preparation were obtained on days 0, 3, 7, 14, 21, 30, and 45. After shaking, a sample was collected and diluted to 1/1000th in water before being analyzed by HPLC.

#### 2.4.3. pH and Organoleptic Aspect

Potential hydrogen (pH) was measured with digital SevenEasy S20 pH meter (Mettler-Toledo, Viroflay, France) equipped with a calibrated InLab^®^ Expert Pro-ISM pH sensor (Mettler-Toledo). A visual inspection was carried out by the examination of each sample under daylight and in front of a white background, under which aspect, color and odor of each sample were noted and after shaking for 30 s for Met suspension study. 

### 2.5. Microbiological Stability Assessment

A microbiological stability study was performed (ACM Pharma, Orléans, France) to evaluate the antimicrobial preservation efficacy of the reconstituted Met formulation. The test was performed as described by the European Pharmacopoeia v. 8.0 for non-sterile products. This test consists of voluntarily contaminating the Met suspension, using a calibrated inoculum of selected microorganisms and counting them after each selected time (0, 14, and 28 days). Six strains of microorganisms were used, namely three fungi (Candida albicans ATCC 10231, Aspergillus brasiliensis ATCC 16404, and Zygosaccharomyces rouxii IP 2021.92) and three bacteria (Pseudomonas aeruginosa ATCC 9027, Staphylococus aureus ATCC 6538, and Escherichia coli ATCC 8739). A neutralizing solution was used to ensure that any preservative effect of the formulation was neutralized for microbial counting. Microorganisms were counted in tryptic soy and Sabouraud agar culture media for bacteria and fungal culture, respectively. 

For each strain, one mL containing appropriate number of colony-forming units (CFU) per mL was added to nine mL of the neutralizing agent, for testing in the absence of the product. One mL of the previous suspension was added into Petri dishes for each medium. This procedure was also employed to the Met formula. CFU number per dish was done after an incubation of five days maximum at 30–38 °C for Trypticase medium, and three days at 20–25 °C for Sabouraud. The method was judged validated when CFU number in one mL of inoculated sample was at least 50% of that obtained in the control (neutralizing solution inoculated with each microorganism). At each time point, the logarithmic reduction in the number of viable microorganisms relative to the value obtained for the inoculum was calculated. This logarithmic reduction was then compared to the values as recommended (see European Pharmacopoeia chapter 5.1.3).

### 2.6. In-Use Acceptability Survey

**Figure 1:**
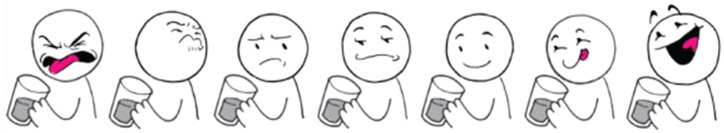
Seven facial children expression presented to estimate taste acceptance: TASTY scale adapted with permission from Ref. [16]. Copyright year 2020, copyright owner’s name the Pediatric Pharmacy Association (PPA).

After one year of Met suspension use, a questionnaire was given to the parents of 7 children (Table S2). To evaluate the appreciation of taste, the TASTY children facial expression scale (Figure 1) was used by the parents [16].

### 2.7. Data Analysis

Data analysis was performed using Excel 365 (Microsoft, Seattle, WA, USA) and Prism (GraphPad Software, version 7.04, San Diego, CA, USA, 2017). Descriptive statistics for continuous variables were expressed as mean ± standard deviation (SD) unless otherwise specified.

## 3. Results

### 3.1. Validation of Stability-Indicating Liquid Chromatography Met Assay

**Figure 2:**
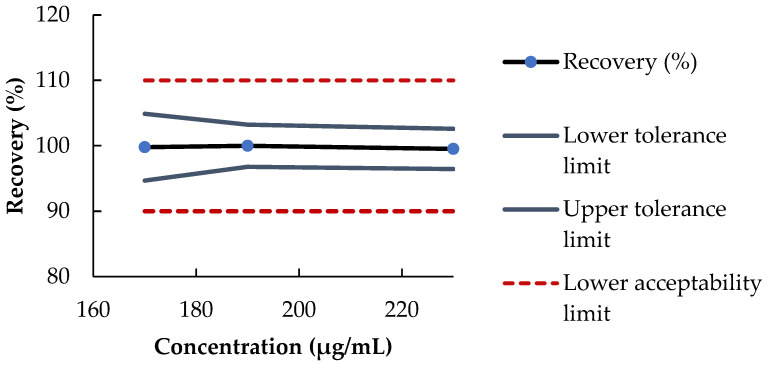
The 95% accuracy profile of the method according to the Met concentration.

Met calibration curve was linear between 160 µg/mL and 240 µg/mL (Table 2) (y = 0.294 (±0.004)x − 1.841 (±1.270); r^2^ = 0.994). The Met retention time was ~6.5 min. Selectivity was assessed (no peak deformation or change in UV spectra in the presence of excipients) and no matrix effect was demonstrated. As shown in Table 3, Met assay precision was ≤1.55% and accuracy was no less than 98% for all Met concentration tested, and the repeatability was 0.6% (*n* = 10). In addition, the accuracy profile was within the acceptability limits of 10% (Figure 2). The LOD and LOQ were 14 µg/mL and 42 µg/mL, respectively, which was acceptable for the concentration range measured.

**Figure 3:**
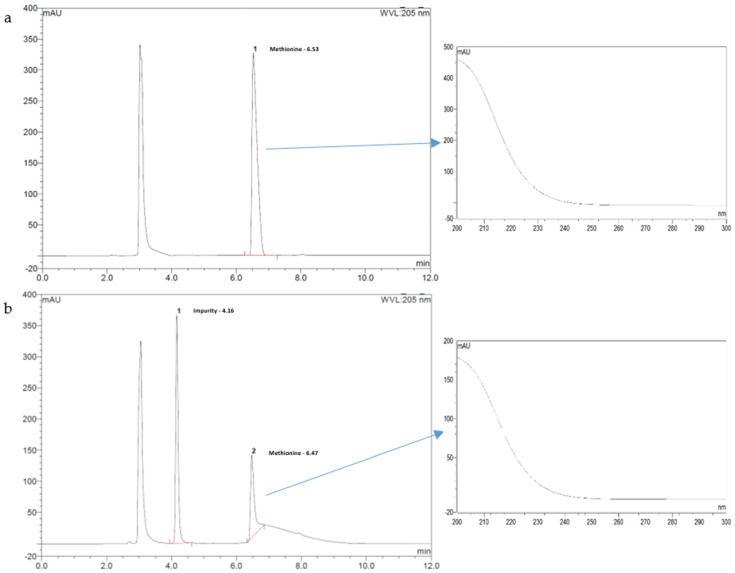
(**a**) Chromatogram of methionine suspension in 1 M HCl less than 15 min after mixing (Met retention time ~6.5 min); (**b**) Chromatogram of methionine suspension in 1 M HCl after 72 h at 70 °C with a degradation product detected at 4.2 min. Insert on the right panel pointed by an arrow: UV spectra of the 6.5 min eluted peak with x axis: wavelength (200–300 nm) and absorbance response y axis (mAU).

The forced degradation study showed a high sensitivity of Met to oxidation and acidic conditions (Table 4). No degradation products coeluted with Met. Indeed, no change in the shape of the Met peak was observed, and the measured UV spectrum (Figure 3) or 3D plot (Figure S2) of the Met peak (200 to 400 nm) was similar to the expected Met spectra. Thus, the developed method indicates good specificity for studying Met degradation and allows its use as a stability indicator method.

### 3.2. Met Formulation Optimisation

The quantities of some excipients were optimized to obtain a better acceptability in terms of odor, taste and uniformity of the content. Regarding odor and taste, a range of fruit flavors were tested in proportions from 1% to 6% (*w*/*v*). Of the fruit flavors tested, the 5% strawberry flavor was found to be satisfactory in masking the Met odor (Table S1). To improve taste, sucralose amount was tested from 0.05 to 0.2% (*m*/*v*). Below 0.1% of sucralose content, the preparation was not sufficiently sweet, while above 0.2% it was judged overly sweet.

The amount of xanthan gum used was studied from 0.1% to 0.5%. The preparation was judged to be overly thick and improper to withdraw it with oral dispenser with an amount of xanthan gum higher than 0.3% (*m*/*v*). A visual inspection of the water reconstituted Met suspension containing xanthan gum at 0.3% showed a homogeneous particle distribution after manual shaking without powder agglomerate/cake over the 45 days of the study.

To assess Met dispersibility of the suspension the Met content was assessed after 10 s of manual shaking with two tests. Three levels of sampling were performed in the bottle Met suspension and analyzed for Met concentration. The mean Met expected concentration was 94.7 ± 5.2%, 93.6 ± 3.4%, and 93.3% ± 2.0%, for bottom, middle and top sample, respectively. Three samples were withdrawn using an oral syringe at three different times after reconstitution. The mean Met expected concentration was 96.2% ± 2.7%; 96.7% ± 0.8% and 99.4% ± 1.0% from 1 h, 5 h, and 3 days, respectively.

Regarding the other excipients, sodium benzoate was added at a regular concentration of 0.3% (*m*/*v*) for its antimicrobial properties at pH below 5, and citrate was added to the formulation for a target pH of 4.5 [17]. The kinematic viscosity of the Met suspension was measured at 416.8 ± 2.2 centiStokes (*n* = 3). The density of the suspension having been measured at 1.02 ± 0.03 g/mL (*n* = 3) the absolute viscosity of the Met suspension corresponds to 425.1 mPa.s.

### 3.3. Physicochemical Stability

#### 3.3.1. Unreconstituted Met Formulation

**Figure 4:**
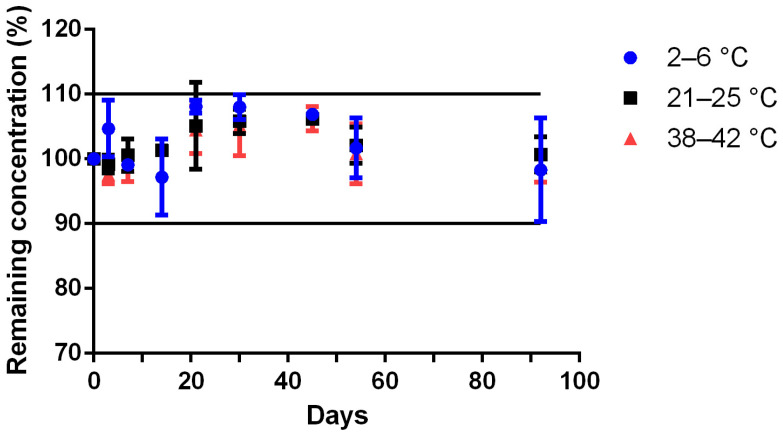
Stability over time of methionine formulated powder at 4 ± 2 °C (blue circle), 23 ± 2 °C (black square), 40 ± 2 °C (red triangle) in glass pharmaceutical bottles and over time. Remaining methionine concentration, calculated by the ratio of the concentration on the tested day to the concentration at day 0; values are mean ± SD (*n* = 3).

The unreconstituted Met powder formulation (Table 1) stored at 23 ± 2 °C, 40 ± 2 °C or 4 ± 2 °C retained its concentration around 100 ± 10% of the baseline value at 92 days after compounding (Figure 4). No color or odor changes occurred in samples stored at 4 ± 2 °C, remaining white and with a strawberry smell. In two out of three samples stored at 23 ± 2 °C, a slightly change in color occurred at 92 days, the powders having taken a very light beige color. In samples stored at 40 ± 2 °C, a change in color and odor occurred at day 14 in all three preparations, with a strong brown color and a heavy unpleasant smell.

#### 3.3.2. Reconstituted Met Formulation

**Figure 5:**
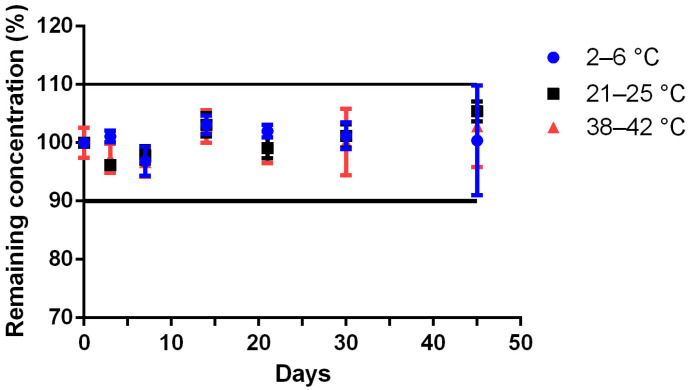
Stability of Methionine 200 mg/mL suspension at 4 ± 2 °C (blue circle), 23 ± 2 °C (black square), 40 ± 2 °C (red triangle) in glass pharmaceutical bottles and over time (remaining methionine concentration, calculated by the ratio of the concentration on the tested day to the concentration at day 0; values are mean ± SD; *n* = 3).

**Figure 6:**
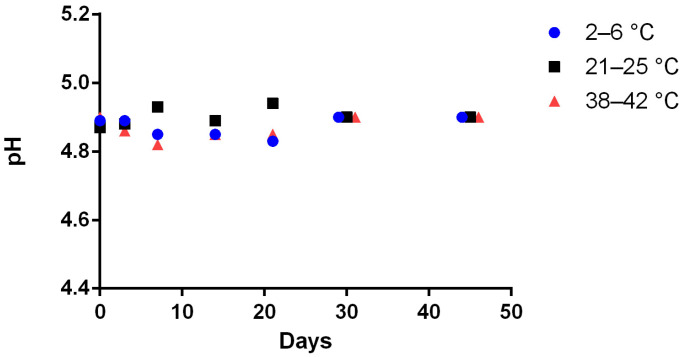
pH value of the reconstituted Methionine 200 mg/mL formulated suspension stored at 4 ± 2 °C (blue circle), 23 ± 2 °C (black square), 40 ± 2 °C (red triangle) in glass pharmaceutical bottles and over time (for more visibility the points have been shifted for days 30 and days 45; standard deviation bars are not visible on the figure due to low data scatter; *n* =3).

The Met formulation (Table 1) reconstituted with 100 mL of water in 200 mL glass bottle type 3, stored between 4 ± 2 °C, 23 ± 2 °C and 40 ± 2 °C retained its concentration around 100 ± 10% of the baseline value at 45 days after compounding (Figure 5). No pH change occurred in any samples stored between 4 ± 2 °C, 23 ± 2 °C and 40 ± 2 °C for the reconstituted at 45 days after compounding (Figure 6). In samples stored between 4 ± 2 °C or 23 ± 2 °C, no color or odor change occurred, with the samples maintaining a white color and a strawberry smell. In samples stored between 40 ± 2 °C, a change in color and odor occurred after 17 days, the samples having taken on a yellowish aspect. 

### 3.4. Microbiological Assessment

The preservative effectiveness of the Met formulation against fungi and bacteria is presented in Table 5. The Met suspensions exhibited adequate preservative efficacy on days 14 and 28 against all six microorganisms, as shown by the measured log reduction for each of them, which meets the criteria of the European Pharmacopoeia (monograph 5.1.3). No growth was observed between day 14 and day 28.

### 3.5. Real-Life Survey: Acceptability of the Met Suspension 

The real-life survey included a panel of seven children treated with Met suspension for at least 1 year, and three patients had—at the time of the survey—a gastrostomy through which all nutritional intakes, including medications, were administered (Table S2). Prior to the use of Met suspension, five patients briefly experienced the use of oral Met treatment prepared in capsules requiring opening and dilution with water. Of these, all parents have preferred the handling of Met suspension over Met capsules and assigned a mean score of 4.7 ± 0.5 out of 5 regarding the ease/preference of using Met suspension. In the four patients without a gastrostomy, the strawberry flavor was considered acceptable and parents assigned a mean score of 3.5 ± 1.7 out of 7 using the child’s facial expression (Table S2). The odor of the Met suspension was rated at 3.1 ± 1.5 out of 5 (Table S2).

## 4. Discussion

Rare ILLD diseases characterized by double homozygous Ala393Thr/Ser576Leu mutations on *MARS1* are particularly severe. Mortality reached 59% with about half of the deaths occurring before the age of two years despite repetitive whole-lung lavages and anti-inflammatory treatments (e.g., steroids) [6]. Rescue of mutated MetRS activity has been demonstrated to be possible by increasing the plasmatic concentration of its physiological substrate [6,7]. Promising results have been recently documented by oral Met supplementation in four children with PAP and multisystemic dysfunction related to MARS1 mutations with full remission of the disease in two patients and a clear ongoing improvement in the two others [6]. The posology of Met is adapted to the total body weight of the child, and according to the results of the Met plasma level. A gradual increase in doses is carried out at the start of treatment and throughout the child’s development [6]. Met supplementation as a liquid pharmaceutical form is particularly interesting for facilitating dose adjustments and infants’ acceptance.

To this end, we developed a 200 mg/mL Met bulk powder. An oral Met suspension is easily obtained after water reconstitution by caregivers or family. A stability indicating analytical assay was validated for measuring Met to ensure the stability validation of pharmaceutical Met formulation. The Met assay developed fulfills analytical and ICH requirements (i.e., linear, precise, and accurate), and was shown specific for Met stability assays as no interference peaks were detected as demonstrated by the forced Met degradation studies performed with the formula containing excipients as detailed in Table 1.

Acceptability of drugs is crucial for children due to inherent characteristics of this population including swallowing difficulties and strong taste sensitivity [18,19,20]. Acceptability as defined by drug agencies includes the pharmaceutical characteristics of the medicine such as palatability, size and shape [21]. Palatability is defined as the overall appreciation of a medicine towards its smell, taste, aftertaste and texture. The attractiveness of pediatric medicinal products should be also carefully balanced between the risk of inadequate patient acceptability and accidental intake and should be discussed with regards to all aspects of the medicine, the dosage form, the formulation, and the strength, as well as the primary and any secondary drug packaging. Unless otherwise justified, the palatability of a paediatric preparation should be satisfactory on its own merit. 

In terms of palatability, Met is a sulfur amino acid with a strong and unpleasant smell and taste [10]. Options that can be taken to improve the palatability of a paediatric formula include a judicious choice of excipients, including taste masks, sweeteners and flavouring agents. Paediatric preparations must not become too attractive to children as this is known to increase the rate of accidental poisoning [22]. In this regard, sucralose and several flavoring agents were tested. Only strawberry in combination with sucralose was judged acceptable and made it possible to partially mask the bad taste of Met. In addition, a pharmaceutical powder for reconstitution exhibits a reduced risk of massive ingestion, and a secure childproof cap is also present on the bottles. It is also important to consider excipients that are safe for this very young paediatric population, as this treatment could be administered very early or even at birth. Indeed, concerns are particularly raised against parabens, which are among the most used preservatives in medicines [23]. In this regard, sodium benzoate was chosen as preservative in this formulation, the age of the treated population being over 56 days old [24,25]. 

Critical product quality attributes to be considered for oral suspensions include physicochemical characteristics of the solution such as viscosity, potential for foaming, air entrapment, sedimentation, and sticking of the suspended active substance to the primary container and to the measuring device. Where particles sedimentation cannot be avoided, easy re-suspension with moderate shaking is required to reduce the risk of dosing errors due to inhomogeneous distribution of the active substance. The risks of under- and overdosing to the child because of inadequate shaking should be considered. In view of the relatively low water solubility of Met (~56 mg/mL) [26], an oral suspension was formulated. To ensure a fairly good dispersibility of the suspended Met particles when taking the medication, as well as to reduce the risk of pulmonary aspiration when administering the suspension in the child’s mouth, thickeners/suspending agents have been added in the formula. The homogeneity of the suspension has been checked by the determination of Met content at the top, middle and bottom of the suspension, and after resuspension by 10 s of gentle manual bottle shaking. The uniformity of the Met content was found to be acceptable as no difference was found in the Met concentration depending on the sampling height site within the bottles, and with no less than 93% of the expected Met concentration. Furthermore, the suspension appears visually homogeneous without powder agglomeration after reconstitution, and the Met concentration was found acceptable with no less than 96% of the expected Met concentration present when withdrawn using an oral syringe after gentle manual bottle shaking before use. Under these conditions, ten seconds of agitation are recommended before an accurate dose for administration can be withdrawn from the reconstituted Met suspension.

The physico-chemical stability study was demonstrated for 92 days at room temperature for the unreconstituted Met bulk powder formulation, and for at least 45 days at refrigerated or ~22 °C storage for the Met suspension. The preliminary results obtained from the stress degradation studies confirmed the sensitivity of Met for oxidation. The need for an antioxidant (e.g., vitamin C) was tested during the accelerated degradation test, without noticeable improvement (Figure S1). The physico-chemical stability of the powder as well as of the suspension was satisfactory already without any specific antioxidant excipient. The microbiological criteria of the European Pharmacopoeia have been respected, which means that the Met preparation can be used without any risk of contamination if the storage conditions are followed. With this information, the pharmacy department can prepare the reconstituted formulation of Met and dispense it for inpatients on pediatric wards or for adults who cannot swallow a solid form. A measuring cup for water measurement as well as an instruction manual will also be provided to parents/caregivers to explain reconstitution and storage of the Met treatment.

A survey was given to the parents to evaluate acceptability of the Met formulation. It showed that all parents were satisfied with this Met suspension. Indeed, the survey indicated that the taste and smell of Met were improved. The taste and odor masking of Met could probably have been modified by the use of other strategies such as cyclodextrin or liposomal complexes. However, these strategies do not dispense with the addition of a flavoring which, in children, seems to be mandatory for a better oral acceptance, especially if the treatment is of long duration. Moreover, the choice was made to develop a formulation that could be more easily transposed to other pharmacy departments and without making the formulation too attractive (candy type), as the risks of overdose in Met are not well known [27].

## 5. Conclusions

This study allowed the validation of an original formulation of Met in the form of an oral powder to be reconstituted. It was optimized in terms of odor and taste by using ingredients such as sucralose and strawberry flavors. Adequate dispersibility of Met in the suspension was ensured by the addition of thickeners. The stability study showed 92-day stability at 4, 23, or 40 °C for the bulk powder. Once reconstituted with water, microbiological and physicochemical studies confirm the stability of the suspension for at least one month. This pharmaceutical form appeared to be more suitable in terms of treatment administration and patient acceptability for the pediatric population, as shown by the survey conducted after at least one year of treatment. Indeed, it facilitates the gradual adaptation of dosages over time and the administration of the treatment by the parents to the children.

## Figures and Tables

**Table 1:** Composition of Methionine formulation for oral suspension (200 mg/mL).

Ingredients	Amount of Pure Powder (mg) for 100 mL
L-Methionine	20,000
Strawberry flavor	5000
Sodium citrate	1730
Sodium carmellose	1000
Citric acid	860
Sodium benzoate	300
Xanthan gum	300
Sucralose	100

**Table 2:** Precision and accuracy of the calibration curve.

Theoretical Concentration (µg/mL)	Calculated Mean Concentration ± SD (µg/mL; *n* = 3)	RSD (%)	Mean Accuracy ± SD (%)
160	159.1 ± 1.9	1.2	99.4 ± 1.2
180	179.7 ± 2.0	1.1	99.8 ± 1.1
200	202.4 ± 2.3	1.1	101.2 ± 1.2
220	219.7 ± 1.8	0.8	99.9 ± 0.8
240	239.1 ± 2.8	1.2	99.6 ± 1.2

SD: Standard deviation; RSD: relative standard deviation.

**Table 3:** Intra and inter-day precision and accuracy results at three concentration levels.

		Intra-Day Validation			Inter-Day Validation		
Theoretical Concentration (µg/mL)	Day	Mean Calculated Concentration ± SD (µg/mL) (*n* = 3)	RSD (%)	Accuracy (%)	Mean Calculated Concentration ± SD (µg/mL) (*n* = 9)	RSD (%)	Mean Accuracy ± SD (%)
170	1	166.8 ± 1.2	0.7	98.1	169.6 ± 2.6	1.6	99.8 ± 1.6
	2	170.0 ± 0.2	0.1	100.1			
	3	172.1 ± 0.2	0.1	101.2			
190	1	190.5 ± 4.2	2.2	100.3	190.0 ± 2.1	1.1	100.0 ± 1.1
	2	191.8 ± 0.1	0.1	100.9			
	3	187.7 ± 1.1	0.6	98.8			
230	1	228.1 ± 4.8	2.1	99.2	228.9 ± 2.4	1.0	99.5 ± 1.0
	2	231.6 ± 0.1	0.1	100.7			
	3	227.0 ± 1.9	0.8	98.7			

Standard deviation (SD); Relative standard deviation (RSD).

**Table 4:** Methionine Forced Degradation Study.

Stress Conditions and Time of Analysis	% of Met Remaining	Retention Time of the Detected Degradation Product(s) (min)
Acidic (1 M, 70 °C, H72)	60.9	4.2
Alkaline (1 M, 70 °C, H72)	90.4	4.2 and 5.25
Oxidative (0.03%, 70 °C, H0; H3)	68.5 (H0); 1.0 (H3)	No degradation peak detected
Photolysis (H32)	84.3	3.1

Immediate (H0), 3 h (H3), 32 h (H32) or 72 h (H72) after mixing Met suspension and the stress agent.

**Table 5:** Results of Antimicrobial Effectiveness.

Microorganism	Inoculum Log_10_	Quantification Day 14 CFU/mL
Escherichia coli	5.5	<10 (>4 Log reduction)
Pseudomonas aeruginosa	5.8	<10 (>4 Log reduction)
Staphylococus aureus	5.6	<10 (>4 Log reduction)
Candida albicans	5.6	<10 (>4 Log reduction)
Aspergillus brasiliensis	5.7	<10 (>4 Log reduction)
Zygosaccharomyces rouxii	5.6	<10 (>4 Log reduction)

Inoculum: count of microrganisms introduced per mL of product. The Log equivalence of the result is indicated.

## Data Availability

All main data are available in the manuscript.

## Supplementary Materials

The following are available online at https://www.mdpi.com/article/10.3390/pharmaceutics15030957/s1, Figure S1: Percentage of Methionine (Met) degradation less than 15 min after reaction with H_2_O_2_ 0.3% or H_2_O_2_ 0.03% without (control), and with 0.1% or 1% of vitamin C (Vit C), Figure S2: (a) Example of Chromatogram and 3D-plot (200–300 nm) of methionine suspension in 0.1 M HCl less than 15 min after mixing (Met retention time ~6.5 min). (b) Chromatogram and 3D-plot (200–300 nm) of methionine suspension in 0.1 M HCl after 72 h at 70 °C with a degradation product detected at 4.2 min, Table S1: Result of the study of aromas on a panel of five adults using a subjective scale from 1 to 5; 5 being the best score, Table S2: Result of acceptability parents survey after at least one year of use by their children.
