# Supplementary material for: Pharmaceutical Oral Formulation of Methionine as a Pediatric Treatment in Inherited Metabolic Disease
Source: Pharmaceutics. 2023 Mar 16;15(3):957. doi: 10.3390/pharmaceutics15030957 (PMC10056843; doi:10.3390/pharmaceutics15030957)
Supplement: Supplementary file 1 [file pharmaceutics-15-00957-s001.zip › pharmaceutics-2153418-supplementary.pdf]

pharmaceutics

Supplementary Materials: Pharmaceutical Oral Formulation of Methionine as a Pediatric Treatment in Inherited Metabolic Disease

Benjamin Querin, Arnaud Schweitzer-Chaput, Salvatore Cisternino, Sylvain Auvity, Anne-Sophie Fauqueur, Abdel Negbane, Alice Hadchouel, Joël Schlatter and Camille Cotteret

Table S1. Result of the study of aromas on a panel of five adults using a subjective scale from 1 to 5; 5 being the best score.

Test subject	Litchi flavor	Cotton candy flavor	Orange flavor	Strawberry flavor
A	1	2	2	4
B	1	1	3	5
C	1	2	2	5
D	1	3	1	5
E	1	1	2	4
**Mean**	**1**	**2**	**2**	**5**

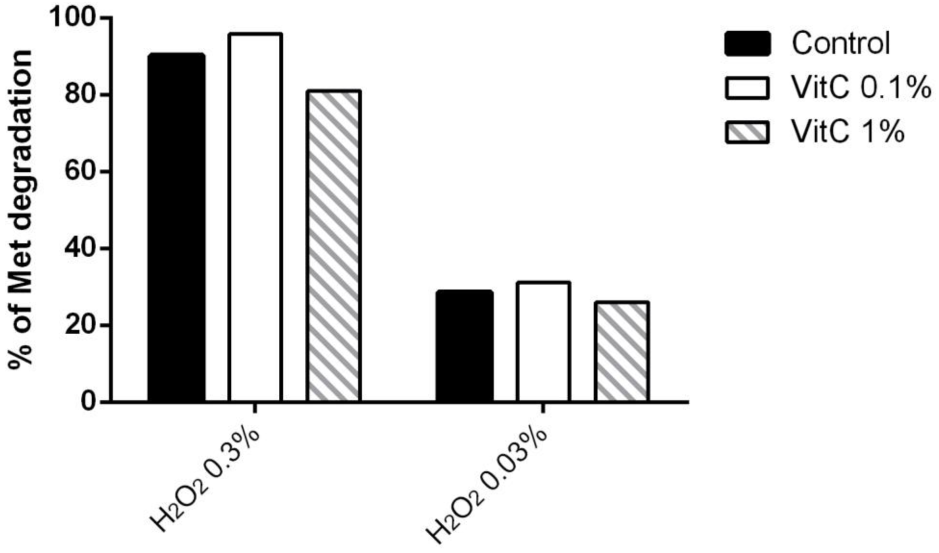

Figure S1. Percentage of Methionine (Met) degradation less than 15 min after reaction with H_2O_2 0.3% or H_2O_2 0.03% without (control), and with 0.1% or 1% of vitamin C (Vit C).

Table S2. Result of acceptability parents survey after at least one year of use by their children.

Item	Survey 1	Survey 2	Survey 3	Survey 4	Survey 5	Survey 6	Survey 7	Results (mean ± SD)
Age	4 years 4 months		2 years 11 months	2 years 3 months	3 years 9 months	20 months	6 years	
How long has your child been taking the methionine syrup? (month)	12	12	13	17	14	15	12	13.6 ± 1.9
Was your child taking methionine capsules before using the syrup?	Yes	Yes	Yes	No	Yes	No	Yes	
If so, do you prefer methionine syrup or capsule?	Syrup	Syrup	Syrup	-	Syrup	-	Syrup	
If so, on a scale of 1 to 7 symbolized by the facial expressions below, how would you rate your child's enjoyment of methionine capsules? Give a score out of 5, where 1 is the worst score	-	-	-	-	1	-	5	
Are you satisfied with the ease of use of the syrup? Give a score out of 5, where 1 is the worst score	4	4	5	5	5	5	5	4.7 ± 0.5
How would you rate the smell of methionine syrup? Give a score out of 5, where 1 is the worst score	3	3	5	3	2	1	5	3.1 ± 1.5
Is Strawberry Taste acceptable or not for your child?				Yes	Yes	Yes	Yes	
Does your child take a drink or food immediately after taking the methionine syrup to eliminate the taste of the syrup?	Gastrostomy intake	Gastrostomy intake	Gastrostomy intake	No	Yes	Yes	Yes	
If yes, on a scale of 1 to 7 symbolized by the facial expressions below, how would you rate your child's appreciation of methionine syrups?				2	3	6	3	3.5 ± 1.7

a

b

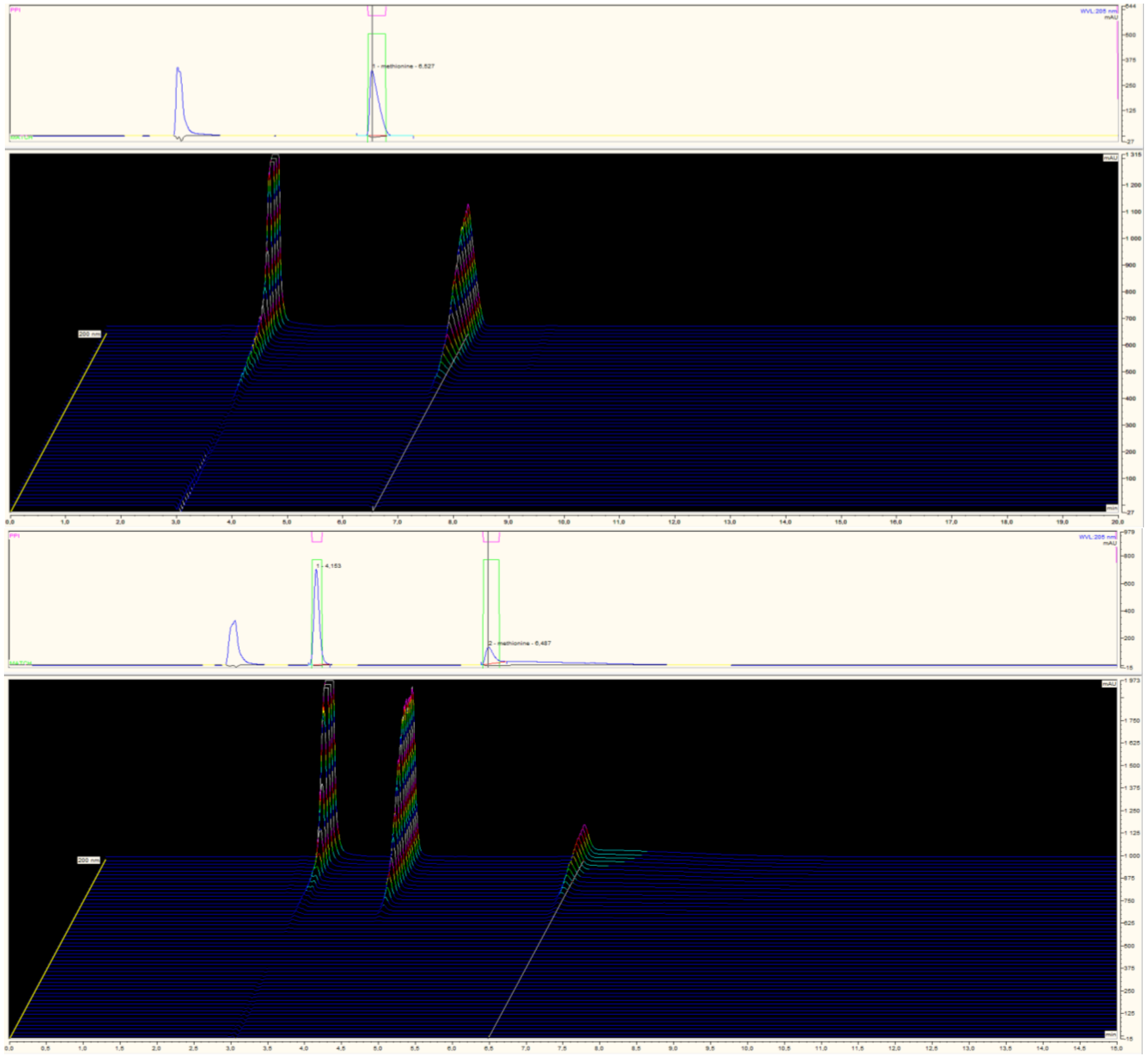

Figure S2. (a) Example of Chromatogram and 3D-plot (200–300 nm) of methionine suspension in 0.1M HCl less than 15 minutes after mixing (Met retention time ~6.5 min). (**b**) Chromatogram and 3D-plot (200–300 nm) of methionine suspension in 0.1M HCl after 72h at 70°C with a degradation product detected at 4.2 min
